# Complexities of nursing in healthcare waste management in hospitals

**DOI:** 10.1590/0034-7167-2023-0391

**Published:** 2024-12-16

**Authors:** Maria José Carvalho Ferreira, Carla Aparecida Arena Ventura, Glaucia Valente Valadares, Thiago Privado da Silva, Laura Johanson da Silva, Ítalo Rodolfo Silva

**Affiliations:** I Universidade Federal do Rio de Janeiro. Rio de Janeiro, Rio de Janeiro, Brazil; II Universidade de São Paulo. Ribeirão Preto, São Paulo, Brazil; III Universidade Federal do Estado do Rio de Janeiro. Rio de Janeiro, Rio de Janeiro, Brazil

**Keywords:** Waste Management, Nursing, Nursing Care, Hospitals, Sustainable Development., Administración de Residuos, Enfermería, Atención de Enfermería, Hospitales, Movilidad Sostenible.

## Abstract

**Objectives:**

to understand how healthcare waste management is developed by nursing professionals in hospitals.

**Methods:**

qualitative research, whose theoretical and methodological frameworks were Complexity Theory and Grounded Theory. Thirty-two nursing professionals from a public hospital in Rio de Janeiro participated in the study. Semi-structured interviews were used.

**Results:**

nursing affects healthcare waste management multidimensionally. Shortage of materials and work overload were identified as factors that influence professionals’ decision-making and increase the risk of improper waste disposal. The lack of knowledge on the subject also influences nursing practice. However, professionals value actions related to hazardous waste.

**Final Considerations:**

nursing professionals recognize themselves in healthcare waste management and understand the need to develop systemic awareness for sustainable practice.

## RESUMEN

**Objetivos:**

comprender cómo la gestión de residuos sanitarios es desarrollada por los profesionales de enfermería en el ambiente hospitalario.

**Métodos:**

investigación cualitativa, cuyos referentes teóricos y metodológicos fueron la Teoría de la Complejidad y la Teoría Fundamentada. Participaron del estudio 32 profesionales de enfermería de un hospital público de Río de Janeiro. Se utilizaron entrevistas semiestructuradas.

**Resultados:**

la enfermería incide de forma multidimensional en la gestión de residuos sanitarios. La escasez de materiales y la sobrecarga de trabajo fueron identificados como factores que influyen en la toma de decisiones de los profesionales y aumentan la eliminación inadecuada de los residuos. La fragilidad del conocimiento sobre el tema también influye en la práctica de enfermería. Sin embargo, los profesionales valoran las actuaciones relacionadas con los residuos peligrosos.

**Consideraciones Finales:**

los profesionales de enfermería se reconocen en la gestión de los residuos de los servicios de salud y comprenden la necesidad de formar conciencia sistémica para la práctica sustentable.

## INTRODUCTION

Across the planet, there is growing concern about sustainability, which aims, through the conscious consumption of natural resources, to guarantee conditions for the survival and development of current and future generations^(1,2)^. In this context, sustainable development must prioritize global health and people’s quality of life. Thus, we speak of a systemic perspective in which life can only be viable and protected when thought of in its multidimensional logic, based on the connections it establishes with the biological, physical/environmental and sociopolitical dimensions^(3)^. Furthermore, from a human perspective, these dimensions only exist because they are integrated and interdependent. Therefore, it is said that sustainable development is, in itself, a complex phenomenon^(4)^.

For complexity, from theorist Edgar Morin’s perspective, the multiple projections or unfolding of the same complex phenomenon assume similarities among themselves based on what is considered to be the holographic principle, in which the whole contains information about the parts and each part presents data about the whole^(5)^. As a result of the challenges for sustainable development, in a specific field, there is solid waste consumption and generation, with due emphasis on healthcare waste (HCW).

Given the numerical reality of the global population, with its more than 8 billion inhabitants, solid waste and HCW consumption and generation have been growing more and more. This increase is also justified by the technological speed that permeates the health sector and allows more procedures to be carried out, while at the same time driving the need for material resources and waste disposal.

Although they do not reach the highest numerical projection in relation to household waste, waste generated by the health sector is of greater concern. In this regard, services that provide health care and generate hazardous waste, such as infectious biological materials, sharp objects, harmful chemicals and radioactive materials, are considered generators of HCW^(6)^. Among the scenarios that produce HCW, the hospital stands out, given that it forms contexts of intense demand for health procedures, either due to the progressive use of technological equipment and materials or the significant use of disposable supplies that require appropriate disposal. Thus, the challenges related to HCW, especially in hospital settings, are relevant, due to the significant concern with natural resource preservation and public health protection^(7-9)^.

Complexity, from Morin’s perspective, demands that social phenomena be considered in globalizing contexts and, in parallel, in their local perspectives. For this reason, the biological impacts resulting from the harmful actions of HCW are, directly and indirectly, contemplated in global agendas^(4)^, in State policies^(8)^ as well as in HCW management plans^(10)^, the latter of which is a local context. The problem related to HCW involves relevant sociopolitical and economic issues within the scope of the performance of each country’s health system, since there are nations where the HCW generation rate has reached 0.14 kg per day/bed, whereas, in other countries, this mean has reached the mark of 6.10 kg per bed/day^(11)^.

Rethinking consumption patterns and reducing waste generation, in order to ensure that they are sustainable, has been a matter of concern and a declared challenge for all members of the United Nations (UN), materially signaled in the 2030 Agenda for Sustainable Development. In this document, the UN lists the set of 17 Sustainable Development Goals (SDGs)^(12)^. Among them, based on this research, we highlighted the one that aims to ensure sustainable consumption and production patterns (SDG 12), in order to allow actions that aim to significantly reduce waste generation through prevention, recycling and reuse. Thus, other SDGs can also, albeit indirectly, be affected by responsible HCW consumption and generation, such as health and well-being (SDG 3), clean water and sanitation (SDG 6) and climate action (SDG 13).

In the case of HCW, it is also necessary to recognize the health system’s complex nature as a fundamental strategy for the responsible generation and consumption of this waste. By system, we mean the dynamic integration among parts for its full development^(5)^. In health systems around the world, nursing makes up the majority of human resources, and its importance in addressing this reality and so many others is not restricted to the numerical expression of these professionals, but achieves significant prominence, considering the multiple care they provide to patients, which is why global organizations recognize the profession as strategic for achieving part of the SDGs^(13-15)^.

Nursing is, therefore, a strategic profession for achieving sustainable practices related to responsible HCW consumption and generation, especially in hospital settings, both because it is the largest consumer of material resources for health care and because it is the driving force for planning, implementing, supervising and assessing resolutive actions for HCW management. Despite this, scientific data indicate low levels of knowledge and a deficit of practices for preserving the environment and its resources as well as insufficient waste management practices by health professionals^(16)^. Thus, from a complex perspective, it is worth asking: how do nursing professionals perceive, in their professional practices in hospital settings, actions related to HCW management?

## OBJECTIVES

To understand how HCW management is developed by nursing professionals in hospital settings.

## METHODS

### Ethical aspects

The research was approved in August 2021 by the Research Ethics Committee. It met all the requirements of Resolutions 466/12 and 580/18 of the Brazilian National Health Council. To guarantee participant anonymity, their identities were preserved using alphanumeric indications. Thus, throughout the article, they were designated as NUR (nurse) and NT (nursing technicians), followed by the number of their respective interviews.

### Theoretical-methodological framework

Complexity Theory, from Edgar Morin’s perspective, was used to interpret the data and construct concepts. This paradigmatic perspective values the inseparability of the social, biological and economic dimensions of complex phenomena. In this context, sustainability is conceived, in its systemic projection, through connections that it establishes with health, culture, environment and society, whose interactions between each part affect the surrounding environment and also the health professionals and patients themselves^(5)^.

For the analytical process, Grounded Theory (GT) was used, and the constructivist aspect of the “Corbinian” school of the method was adopted, which presents a comparative approach, constant questions, theoretical sampling, elaboration and integration of concepts, composed of three paradigmatic dimensions, namely: conditions; actions-interactions; and consequences^(17)^.

### Study design

This is qualitative and explanatory research that correlates subcategories (principles) around the category (concept) to explain the reality investigated. For the research structure, the EQUATOR network guidelines, such as the COnsolidated criteria for REporting Qualitative research (COREQ), were used.

### Methodological procedures

Participants were characterized based on a form with sociodemographic and professional data. To achieve the research objective, semi-structured interviews were conducted, whose leading questions were: what does HCW management mean to you? How do you perceive HCW consumption and generation in the hospital? How do you perceive yourself as a professional, nursing team member, in relation to HCW consumption and generation in the hospital? Based on the responses, circular questions were developed in order to facilitate substantiated data gathering. The interviews took place in individual meetings, in a private environment, in the study setting itself, at previously agreed times that did not compromise the interviewees’ work activities.

### Study scenario

Data were collected at a federal university hospital in the city of Rio de Janeiro, RJ, which offers several clinical specialties and has approximately 550 beds. The study involved the medical and surgical clinic sectors, considering that, in these places, regardless of the clinical specialties, there is a significant HCW generation from direct and indirect nursing care to patients.

### Data source

This was a convenience sample, in which participants were invited in person to the study setting. No guest refused to participate or any participant withdrew from the study.

Nurses and nursing technicians participated in the study, and comprised two sample groups, respectively. Professionals with at least one year of professional experience in the institution, in direct patient care as a nurse or nursing technician in the medical or surgical clinic sector, were included. Professionals working night shift were excluded, given the understanding that HCW generation in the hospital is more expressive during the day, due to the routine of health care/procedures.

The delimitation of two sample groups was supported by the hypothesis that, although they are part of a single professional category, nurses and nursing technicians may present specificities, both due to deontological aspects and specific knowledge.

### Data collection and organization

Data collection took place between September and December 2021. The interviews were recorded digitally (audio) and lasted an average of 30 minutes each. The researchers conducted a pilot test with three potential participants, and no adjustments to the interview script were identified. The interviews were not repeated with the same participant. Analytical and reflective memos were used throughout the collection and analysis process, a process that occurs simultaneously in GTD. These memos allow formulating hypotheses capable of directing new questions or even new sample groups so that researchers can understand where the phenomenon is rooted^(17)^. Furthermore, memos allowed reflections on the development of concepts, thus helping to understand data theoretical saturation, i.e., when the concept presents theoretical density supported by its respective subcategories/principles^(17)^. It is important to highlight that the process of theoretical saturation was discussed among the researchers before data collection was completed.

Data collection was carried out by two nurse researchers, with expertise in the data collection method and the adopted methodological framework, through skills developed in other research with similar methodological morphology, together with the research group to which they are linked. There was no conflict of interest related to research setting professionals. The researchers have no connection with the research setting.

To facilitate analysis, organize data, store files, map concepts and prepare reports quickly, simply and in a computerized manner, the data were imported into NVIVO^®^ 12 after transcription in Microsoft Office Word^®^ 2016. NVIVO^®^ 12 is a fundamental software for GT studies, as the significant amount of data generated in the analytical process is systematically organized with the software in question^(18,19)^.

### Data analysis

The data were subjected to analysis following the GT coding steps, namely: open; axial; and selective integration. In open coding, the data were segmented into distinct parts and rigorously examined, whose comparison aimed to identify similarities and differences between the initial data (preliminary codes). At this stage, codes are provisional^(17)^.

From comparative data analysis, they were grouped by similarities and differences. From this process, conceptual codes emerged, which are presented as an abstract representation of a fact, object or action that the researcher perceives as significant. The grouping of conceptual codes by similarities gave rise to the analytical category^(17)^.

Axial coding requires an analyst to already have some subcategories constructed, to relate them to their category, in order to then generate more accurate and complete explanations about the phenomenon investigated^(17)^. In the integration phase, the category is refined and developed from the theoretical abstractions of its subcategories.

For this study, the subcategories were ordered based on the paradigmatic model of the constructivist GT branch of the “Corbinian” school of the method^(17)^, which considers three components, namely: conditions, which deal with factors that influence the development of the phenomenon; actions-interactions, which deal with strategies for developing and coping with the problem; and consequences, which signal potential reactions based on the strategies implemented.

## RESULTS

Ten nurses and 22 nursing technicians participated in the study, totaling 32 participants, of which 28 were female and four were male. Ages ranged from 27 to 58 years, with an average of 39 years. Participants’ training time showed an average of 12 years and nine months, and an average working time in the research setting of five years and ten months.

As for participants’ training, of the 22 nursing technicians, two reported having completed higher education in nursing. In the group of nurses, only one reported not having a *lato sensu* graduate degree. However, no participant reported having taken a course in the area or for approaching HCW management.

From the analytical process, the category/concept “The multidimensionality of healthcare waste management in hospital nursing practice” emerged. To this end, the category/concept was structured based on the following subcategories/principles, distributed in the paradigmatic model as follows:

Conditions: subcategory 1: Orders and disorders in healthcare waste generation and disposal in hospital settings; subcategory 2: Risks and uncertainties in healthcare waste disposal.

Actions-interactions: subcategory 3: Material waste management: the non-linear logic of healthcare waste generation and consumption in hospital settings; subcategory 4: From knowledge to action: the pathology of knowledge in nursing professional training and its relations with healthcare waste management.

Consequences: subcategory 5: Products and producers of themselves: the challenge of managing healthcare waste during the COVID-19 pandemic.

It is important to highlight that the excerpts from participants’ statements are merely illustrative of each subcategory, given that they were developed from numerous preliminary codes, resulting from a significant volume of data from transcribed interviews.

### Subcategory 1 - Orders and disorders in healthcare waste generation and disposal in hospital settings

The results revealed that nursing professionals perceive themselves as producing a significant amount of HCW, but consider that it is possible to reduce the generation of this waste through the rational management of material resources without affecting quality of care.

*But we can, yes, reduce the amount of material used, reduce it a little, see what is necessary, use what is necessary in assistance.* (NT01)

Although waste generation in hospital settings is a concern for nursing professionals, reducing the amount of waste generated can be recognized as a measure to promote sustainability perceived by the professionals themselves.

The results also demonstrate that this high productivity of HCW in hospital settings can be influenced by the scarcity of quality material resources for health care. In this context, professionals need to find different ways to deal with the lack of adequate equipment and supplies to provide quality care to patients. Adapting to circumstances and finding creative solutions to overcome the lack of resources, given the limitations faced in the service, were challenges highlighted by nursing professionals in these situations. This reality has led to an increase in HCW generation.

*So, in our reality, we would do, for instance, the dressing, we would use the tub to be able to put in some serum, then do the dressing later* [...] *here we don’t even have an autoclave, so there’s no way to use a tub, a basin, so what’s the deal? It’s* [...] *diapers or compresses, so the production of waste, waste that we wouldn’t produce in this way, if we had the tub, the basin, we end up producing, because there’s no way.* (NT08)

The results also highlighted the need for an accurate list of all materials used in care provision as a way of controlling inputs. In these circumstances, simple work processes, such as specifying the number of gauze packets, sterile gloves and other materials to be used in the various procedures performed by nursing staff, are considered relevant.

[...] *when you report, for instance, the dressing you performed, you have to write down how many gauze packs you used, how many sterile gloves, whether it was serum, whether it was alcoholic chlorhexidine; all of this has to be reported.* (NUR05)

Thus, nursing professionals recognize HCW management as a demand of the work process they perform and that they should strive to reduce the production of these materials. In this regard, in their meanings, they highlight the planning of materials for nursing care as a starting point for sustainable care.

The data also revealed the structural conditions and resources offered by the work context for adequate waste management and the barriers faced by professionals in their daily hospital routine.

Routine and excessive care demands drive an accelerated pace of work and lack of time, which affect decision-making processes, especially those that require agility from professionals, such as segregating material in greater quantities than necessary to perform a certain care, generating waste of materials.

*I think the rush of everyday life, the lack of time* [...] *you go out, I’ll open it, because otherwise I’ll waste time if I leave it to ask someone if I need more.* (NUR07)

The data reveal that the pace of work and the consequent lack of time favor inadequate HCW segregation. Furthermore, professionals indicated that they know that rushing and not paying enough attention when disposing of waste can lead to mistakes, resulting in inadequate and potentially dangerous disposal of materials used in health procedures. This incorrect segregation compromises the effectiveness of the collection, transportation and treatment processes, increasing the risk of environmental contamination and endangering the health of the professionals involved as well as the surrounding community.

*There’s no time to even want to segregate. It’s very hard. There are days when we seem like a robot sometimes.* (NT21)

*You end up doing it wrong, by rushing and saving time. I think it’s always a matter of time, stopping to do things at the right time, I think it greatly reduces the risk of incorrect disposal.* (NT12)

It is important to highlight that the hypothesis that guided the composition of two sample groups of this research, such as nurses and nursing technicians, may present specificities, both due to deontological aspects and specific knowledge, and was not supported by the data, since there were no specificities in the results in relation to the aforementioned groups.

### Subcategory 2 - Risks and uncertainties in healthcare waste disposal

This subcategory reveals, based on nursing professionals’ perception, how, in their own practices, HCW disposal and segregation are carried out. Thus, they revealed inconsistencies in these processes when it occurs according to participants. This reality is complex because it is influenced by several factors, as exemplified in the following excerpts:

*The blood bag, right?! After transfusion, today it goes in the regular trash. From what we know, it could be wrong, first you filter it, but sometimes we have doubts.* (NT18)

*Sometimes, they throw it away unintentionally, because it goes unnoticed, even though it is something important* [...] *everyone insists on the same thing: “discarding needles is in the trash”, but, from time to time, we pick up a needle from the trash.* (NT12)

[...] *I have seen sharp objects being thrown in the common trash, in the infectious trash, things like that that are gross* [...] *disposal, for instance, of material, of fluid from contaminated material in common trash.* (NUR03)

Although common waste accounts for the largest proportion of hazardous waste in the health sector, such as sharps, the latter is the waste that is of greatest concern to nursing professionals. Amid the risks, uncertainties and illusions of the complexity inherent in this situation, professionals surveyed demonstrated that they understand the risks close to them when they indicate the importance of disposing of more hazardous HCW in their work contexts.

*This care is all for us so that we don’t get punctured and we don’t have an accident when segregating this waste, contamination, of what is glass, ampoules, even medication bottles, antibiotics.* (NT18)

Furthermore, among the multidimensional factors related to HCW management, the provision of material resources, infrastructure and organizational flows stood out by the nursing team. In this context, supposedly simple devices and measures, such as the availability of bins for infectious waste segregation and educational strategies based on efficient information for patients and their companions, were indicated as capable of reducing the chances of mixing infectious and common waste, including food scraps and equipment.

*I can’t tell you, I don’t know if there’s a lack of bins, because before all the wards had two bins, one for regular trash, one for hospital trash and one for sharps, but there’s usually no regular trash. We throw the food in with the equipment* [...] *which is where we throw away the serum, equipment, diapers, etc. So, everything ends up together, but, like, it’s in some wards, others have it, I don’t know why, I don’t know what happened.* (NT09)

*Sometimes, not only the technician, not only the professional, but also there are other people who stay in the ward who do not have this knowledge. The companions, for instance, do not have this knowledge* [...] *if they have to throw it in either of the two, for them, that stuff is trash, it’s the trash can, they won’t know.* (NT17)

The aforementioned reality is supported not only by nursing technicians, but also by nurses.

*What we see is that everyone uses both for everything. You open the infectious waste bin, there’s food inside, you know? There’s just not something that should be in there, so we see that it doesn’t work in the ward.* (NUR05)

*Look, here at the hospital, despite there being white bins, with segregation of infectious and non-infectious waste, I think they are not very good* [...] *segregation is not good, because when you open the bin for the infectious waste, everything is there* [...] *there is food, diapers, a bottle of water.* (NUR08)

Another factor related to the multidimensionality of HCW management is nursing professionals’ knowledge regarding the Healthcare Waste Management Plan.

*I don’t know the Plan* [Healthcare Waste Management Plan]. *Here, what we learned was in practice.* (NT14)

In addition to issues related to knowledge, professionals’ attitudes are also recognized as an intervening condition for nursing’s challenges towards HCW management. In the meantime, the lack of encouragement for the segregation of recyclable materials influences attitudes in favor of sustainable practices.

[...] *I didn’t receive any guidance that there is a place to dispose of disposable waste, no! For instance, the water bottles that we use* [...] *can be recycled and still go into the normal trash, and that’s discouraging.* (NUR08)

### Subcategory 3 - Material waste management: the non-linear logic of healthcare waste generation and consumption in hospital settings

This subcategory demonstrates how waste generation is influenced by hospital material waste management. Furthermore, participants considered that nursing care without resource planning can trigger the inappropriate use of supplies and, therefore, increase waste generation. For nurses, HCW generation is inevitable, but waste is manageable.

*I noticed today, right?! Gauze, for instance, a package of gauze is opened, and then they took everything and threw it away, because it was already open.* (NUR05)

*The other day, in a ward that was closed due to some technical problem, patients were sent to other floors, but there was so much stuff in the ward, about seven oil cans, everything was going to be thrown away, what was I going to do? I can’t put it on another patient. So, that put an end to this waste.* (NUR01)

For nursing professionals, as they need to improvise due to the scarcity of material resources, the measures adopted generate more waste, given the need to use a greater quantity of supplies that are then adapted to provide the best possible care for patients. Thus, the data indicated that the supposed savings in material resources manifested in the deficit of these actually present economic aggravating factors due to the improvisation required for care practices. Due to this reality, the impacts on the environment and on health professionals’ and patients’ health are aggravated.

*So, when there is the right material, no, but when the hospital is short of it, we have to improvise with other things, and that’s when waste occurs. If we have the right material, we don’t waste it.* (NT10)

HCW does not always originate from direct patient care. A recurring finding from the survey indicated that nursing professionals point out the excessive waste of A4 paper, for instance.

*There are more and more printed documents, which we didn’t have here and which should have decreased with the advancement of technology. Sometimes, for instance, a paper appears for every patient who is going to be referred for some type of exam. Before, we would do the referrals in a rush, now they invented a transfer sheet, so there is one more paper for every patient who goes somewhere. So, if we have 27 patients, we did about ten transfers. That’s ten papers, you know? I think it’s absurd, we have to think not only about the cost, but also about waste production.* (NUR02)

Therefore, material waste management was considered an important practice that should be learned in nursing professionals’ daily hospital routine, in order to enable positive measures in the health economy in line with sustainable practices.

### Subcategory 4 - From knowledge to action: the pathology of knowledge in nursing professional training and its relations with healthcare waste management

This subcategory reveals that specialized technical knowledge and practical skills are prerequisites for managing HCW. To this end, nursing professionals considered that their team must be prepared to handle various types of waste, such as sharps, infectious materials, chemicals, medications, among others that may pose a risk to their own health, to patients’ health, and to the environment.

Although standards and legislation require that nursing professionals be trained in appropriate practices for managing HCW, promoting safety and effectiveness in its management, the results revealed insufficient knowledge, which is attributed to the scarcity of training offered on the subject. For participants, these measures can influence them in the identification and correct classification of waste generated by health services, following national and international standards and regulations.

*They talked about waste management, but I think that in the training of a cleaning company they talked about it, but very little. Even the courses don’t teach it. I come from a very old time.* (NT02)

The complex nature of results led to the understanding that nursing know-how in managing HCW is also influenced by the inclusion or absence of environmental issues in the curricula, both in high school and higher education. For study participants, little or no type of approach to the subject was part of their professional training. Despite the above, nursing practice aimed at sustainable practices related to the process of generating and consuming HCW must be based on technical knowledge about the types of waste, their characteristics and classification as well as skills that allow handling these materials safely and preventing risks to health and the environment.

### Subcategory 5 - Products and producers of themselves: the challenge of managing healthcare waste during the COVID-19 pandemic

The results of the research were contextually relevant to the historical moment of facing the COVID-19 pandemic, a reality that also influenced, according to study participants, HCW generation. Thus, with increased demand for health services, there was also a significant increase in waste generation, which made the management of these materials even more challenging.

*Concern about PPE has increased, but so has the use of materials, gowns, gloves, masks* [...] *materials that were more important in COVID-19 have increased significantly due to demand.* (NT06)

Nursing professionals highlighted that the pandemic period has increased a practice that already occurs in the daily work routine in the health area. In that situation, however, the waste and improper disposal of personal protective equipment, supplies and other resources were even more significant, according to nursing team members.

*I think there was a lot of waste* [...] *lack of knowledge about the disease, nobody knows about something new, they still don’t know about it, right?! Everyone was caught unprepared, so there was a lot of waste of material, we see a lot of things.* (NT04)

*PPE use was intensified and there was also a lot of improper disposal, not only in hospitals, but everywhere you could see masks thrown in inappropriate places, for instance.* (NUR09)

According to the data presented by participants and from the perspective of complexity that guided this study, the recent pandemic has only revealed the importance of a systemic awareness that goes beyond the context of healthcare professionals and affects the entire society. Thus, the anticipated consequence, based on what the data in the previous subcategories indicated, seems to revolve around the importance of contextualized civic education capable of recognizing and valuing the need for responsible practices in waste consumption, generation and disposal.

## DISCUSSION

Nursing professionals’ perspective on how they are involved in HCW management supports the understanding that hospital settings are the focus of attention for the problem involving HCW generation, and this reality may present specificities in relation to the institutional management policies/cultures of hospitals, as indicated by the study carried out in private hospitals, whose data indicated the generation of 8.22 (6.39-10.02) kg/bed/day, demonstrating that they tend to be greater generators than public hospitals^(20)^.

In the sociopolitical and economic context, it is known that Brazil is a continental, developing and industrialized country, and this configuration should also be considered when it comes to HCW generation, especially when considering the object of this study, with nursing being perceived as an important intervening factor for HCW management, since it is, in accordance with other global realities, the main contingent of human resources in the health sector. In this regard, it is clear that the mean HCW generated in high-income countries generally varies between 2 and 4 kg/bed/day, which is lower compared to upper-middle and low-income countries, ranging between 4 and 6 kg/bed/day, which may be due to the fact that high-income countries have better management policies, more advanced disposal technologies as well as competent regulatory authority and trained workers compared to lower-middle-income countries^(21)^.

Given the above, as indicated by the recursive-circuit principle of complexity, whose process guides that products and effects are, in parallel, causes and producers of that which produced them^(5)^, it is clear that the relevance of nursing in health systems can form a valuable driving force towards appropriate conduct for HCW management, especially based on appropriate measures to reduce HCW generation in hospital settings in their daily operations, such as systematic control of inputs, with information on the volume of HCW generated by health professionals^(22)^. However, in accordance with the recursive-circuit principle^(5)^, HCW management can be compromised when nursing does not take over its leading role in this process. However, according to the data from our research, this reality does not necessarily depend only on nursing, but also on working conditions, which are affected by the overload of professionals and the deficit of material resources.

Infrastructure was identified as a factor that influences HCW management. Hence, regarding the conditions found for HCW disposal, it was revealed in a survey that the majority of respondents, 358 (90.86%) from health centers and 133 (96.38%) from hospitals surveyed, indicated that their facilities had to segregate containers for hazardous and non-hazardous waste; and 61 (15.48%) of respondents from health centers and 29 (21.01%) from hospitals indicated that HCW containers were not clearly marked or labeled. In the same study, 241 (67.3%) used existing bins to dispose of waste in public health facilities, and 32 (23.19%) of hospitals indicated that HCW containers were not located in appropriate places where they could be needed^(23)^.

Regarding recyclable waste segregation in hospitals, the literature indicates the need to implement solid waste management policies that establish appropriate guidelines and procedures for the segregation, collection, transportation, storage, treatment and final disposal of the waste generated^(24,25)^. The generation of waste destined for recycling, according to research data, was 0.72 (0.45- 1.02) kg/bed/day, which accounts for only 12% of the total general waste and demonstrated how good waste management allows implementing selective collection in hospital settings^(22)^.

Constantly assessing the number of materials used in procedures, considering possible reductions without affecting quality of care, as well as the use of reusable materials, replacing disposable materials with reusable ones, can help save money and reduce the amount of waste generated. This reality can lead to, for instance, the use of reusable plates, cups and cutlery instead of disposable ones, together with the encouragement for implementing a recycling program that enables segregating recyclables, strengthens continuing health education and addresses the issue from a systemic perspective. In addition to this, it is essential to control the stock of materials that favor the reduction of excessive or insufficient purchases for services as well as the reduction of the disposal of expired medications^(22)^.

Another aspect highlighted in the study that influences HCW management is knowledge about this topic. Thus, regarding knowledge of the existence of the Healthcare Waste Management Plan, the research cites that 71.4% of nurses and 40% of nursing technicians claim not to know whether the institution has such a Plan. This rate is even lower when compared to nurses’ knowledge about the existence of a Hospital Infection Control Committee (HICC) as well as an Internal Accident Prevention Committee (IAPC): 86% of technicians and 85% of nurses^(24)^.

The fragility of training nurses and nursing technicians, pointed out by study participants, in relation to knowledge about HCW management, suggests a direct relationship with the need to consider an education that values complexity throughout nurses’ training process as well as in practices related to continuing education in health. To this end, the data reinforced the need for educational and health institutions to provide strategies that contextualize environmental sustainability in perspectives close to the nursing team’s professional practice.

Although humanity has gone through the greatest global health crisis in recent years, with the COVID-19 pandemic, this recent reality has demonstrated, on a large scale, the need for better investment in professional training and due attention from governments and educational and health institutions to environmental issues, notably those related to responsible waste consumption and disposal.

Still regarding the recent pandemic period, it is clear that this context has increased inadequate practices for HCW consumption, generation and disposal as well as deficits in material resources and infrastructure related to this problem, in parallel with the expansion of patient hospitalization flows. In this context, personal protective equipment used by health professionals to treat sick patients and mandatory preventive safety measures, such as face masks and gloves, have led to a substantial increase in waste accumulation worldwide. In China, for instance, around 240 tons of HCW were discarded daily during the pandemic; previously this amount corresponded to 40 tons on normal days^(26)^. However, the problem related to excessive waste generation does not only arise from global health crises, but also from the natural process of population growth and the consequent demand for material resources, waste consumption, generation and disposal. In this context, for instance, even before the COVID-19 pandemic, HSW generation in India had increased from 559 to 613 tons/day between 2017 and 2019, and, during the fight against the pandemic, the country generated around 850 tons of HSW per day^(27)^.

### Study limitations

The reality investigated may have influenced participants’ systems of meaning in relation to HCW management, especially those related to material resources, infrastructure and organizational culture conditions. This is because university hospitals may present specificities not observed in private hospitals, for instance. From an epistemological perspective, the importance of validating the results with experts, as occurs in some studies with GT, is declared as a limitation of this study.

### Contributions to nursing, health or public policy

Although nursing professionals recognize that they are capable of influencing HCW reduction without, however, affecting quality of care, they highlighted the importance of work processes that allow recognizing the multidimensionality involved in HCW management. Furthermore, from the nursing perspective, the study indicates that the deficit of material resources drives work processes based on creative improvisations to supply these resources. This reality, added to the overload of the workers investigated, drives HCW generation.

The study indicates nursing knowledge fragmentation, from the perspective of professionals in the area, in relation to knowledge about HSW management. However, participants emphasize that technical-specialized knowledge and practical skills are conditions for HSW management. In this context, nursing professionals identified that the fragility of knowledge on the subject may result from the lack of approaches in regular training (undergraduate studies) as well as in health services (training). This reality shows the importance of valuing the topic related to HSW management nursing professional training.

## FINAL CONSIDERATIONS

HCW management, in hospital settings, is carried out by nursing professionals from a complex perspective, whose dimensions involve intervening factors for its development, such as strategies and consequences that range from professional and patient safety to population health and environmental preservation.

The scarcity of material resources, associated with the need to improvise with available supplies, was identified as a reality that influences excessive HCW generation. Furthermore, excessive care demands, associated with accelerated pace of work, affect decision-making processes and, therefore, increase the chances of errors by professionals due to material waste and inadequate HCW disposal. Despite this, nursing professionals demonstrated an understanding of the risks close to them when they indicate the importance of disposing of more dangerous HCW in their work contexts.

For the nursing team, their practice related to HCW management is influenced by the inclusion or absence of environmental issues in their training processes as well as by the lack of opportunities to discuss HCW in actions related to continuing health education.

Nurses and nursing technicians recognize that the COVID-19 pandemic has only intensified a chronic problem related to HCW consumption, generation and disposal, so they recognize the importance of a systemic awareness capable of valuing the importance of responsible and sustainable practices in relation to HCW.
