# Supplementary material for: Complexities of nursing in healthcare waste management in hospitals
Source: Rev Bras Enferm. 2024 Dec 16;77(6):e20230391. doi: 10.1590/0034-7167-2023-0391 (PMC11654508; doi:10.1590/0034-7167-2023-0391)
Supplement: Translation

# Translation: Complexidades da enfermagem no gerenciamento dos resíduos de serviços de saúde no contexto hospitalar

**DOI:** 10.1590/0034-7167-2023-0391pt

**Authors:** Maria José Carvalho Ferreira, Carla Aparecida Arena Ventura, Glaucia Valente Valadares, Thiago Privado da Silva, Laura Johanson da Silva, Ítalo Rodolfo Silva

**Affiliations:** I Universidade Federal do Rio de Janeiro. Rio de Janeiro, Rio de Janeiro, Brasil; II Universidade de São Paulo. Ribeirão Preto, São Paulo, Brasil; III Universidade Federal do Estado do Rio de Janeiro. Rio de Janeiro, Rio de Janeiro, Brasil

**Keywords:** Gerenciamento de Resíduos, Enfermagem, Cuidados de Enfermagem, Hospitais, Desenvolvimento Sustentável.

## Abstract

**Objetivos:**

compreender como o gerenciamento de resíduos de serviços de saúde é desenvolvido por profissionais de enfermagem no ambiente hospitalar.

**Métodos:**

pesquisa qualitativa, cujos referencias teórico e metodológico foram a Teoria da Complexidade e a Teoria Fundamentada nos Dados. Participaram do estudo 32 profissionais de enfermagem de um hospital público do Rio de Janeiro. Foram empregadas entrevistas semiestruturadas.

**Resultados:**

a enfermagem afeta o gerenciamento de resíduos de serviços de saúde de forma multidimensional. Escassez de materiais e sobrecarga de trabalho foram apontados como fatores que influenciam a tomada de decisão dos profissionais e potencializam descarte inadequado de resíduos. A fragilidade de conhecimentos sobre a temática também influencia a prática da enfermagem. Entretanto, os profissionais valorizam ações relacionadas aos resíduos perigosos.

**Considerações Finais:**

os profissionais de enfermagem se reconhecem no gerenciamento de resíduos de serviços de saúde e compreendem a necessidade da formação de uma consciência sistêmica para uma prática sustentável.

## INTRODUÇÃO

Em todo o planeta, é crescente a preocupação com a sustentabilidade, que visa, a partir do consumo consciente de recursos naturais, garantir condições para a sobrevivência e o desenvolvimento das gerações atuais e vindouras^(1,2)^. Nessa conjuntura, o desenvolvimento sustentável deve primar pela saúde global e qualidade de vida das pessoas. Assim, fala-se de uma perspectiva sistêmica na qual a vida só pode ser viável e protegida quando pensada em sua lógica multidimensional, a partir das conexões que estabelece com as dimensões biológicas, físicas/ambientais e sociopolíticas^(3)^. Ademais, sob perspectiva da humanidade, essas dimensões apenas subsistem por serem integradas e interdependentes. Por isso, diz-se que o desenvolvimento sustentável é, em si, um fenômeno complexo^(4)^.

Para a complexidade, sob a vertente do teórico Edgar Morin, as múltiplas projeções ou desdobramentos de um mesmo fenômeno complexo assumem semelhanças entre si a partir do que considera ser o princípio hologramático, em que o todo contém informações das partes e cada parte apresenta dados sobre o todo^(5)^. Como desdobramento dos desafios para o desenvolvimento sustentável, em um campo específico, tem-se o consumo e a geração de resíduos sólidos, com o devido destaque para os resíduos de serviços de saúde (RSS).

Diante da realidade numérica da população global, com os seus mais de 8 bilhões de habitantes, o consumo e a geração de resíduos sólidos e, por conseguinte, de RSS, têm crescido cada vez mais. Esse aumento também é justificado em decorrência da celeridade tecnológica que atravessa o setor de saúde e permite que mais procedimentos sejam realizados, ao mesmo tempo que impulsionam a necessidade de recursos materiais e descarte de resíduos.

Embora não alcancem a maior projeção numérica em relação aos resíduos domésticos, os resíduos gerados pelo setor de saúde são mais preocupantes. Nesse sentido, os serviços que oferecem assistência à saúde e geram resíduos perigosos, como materiais biológicos infecciosos, objetos cortantes, produtos químicos nocivos e materiais radioativos, são considerados geradores de RSS^(6)^. Entre os cenários produtores de RSS, destaca-se o hospital, haja vista conformar contextos de intensa demanda de procedimentos de saúde, seja em decorrência da progressiva utilização de equipamentos e materiais tecnológicos ou da expressiva utilização de insumos descartáveis que requerem destinação apropriada. Desse modo, os desafios relacionados aos RSS, com destaque para o contexto hospitalar, são relevantes, devido à preocupação significativa com a preservação dos recursos naturais e a proteção da saúde pública^(7-9)^.

A complexidade, na perspectiva de Morin, pede para que os fenômenos sociais sejam pensados em conjunturas globalizantes e, de modo paralelo, em suas perspectivas locais. Por essa razão, os impactos biológicos decorrentes das ações nocivas dos RSS são, direta e indiretamente, contemplados em agendas globais^(4)^, em políticas de Estado^(8)^, bem como em planos de gerenciamento de RSS^(10)^, esta última de conjuntura local. A problemática relacionada aos RSS envolve relevantes questões de ordem sociopolítica e econômica no âmbito do desempenho do sistema de saúde de cada país, pois há nações em que a taxa de geração de RSS chegou a 0,14 kg por dia/leito, enquanto que, em outros países, essa média alcançou a marca de 6,10 kg por leito/dia^(11)^.

Repensar os padrões de consumo e reduzir a geração de resíduos, de modo a assegurar que esses sejam sustentáveis, tem sido objeto de preocupação e desafio declarado para todos os membros da Organização das Nações Unidas (ONU), materialmente sinalizado na Agenda 2030 para o Desenvolvimento Sustentável. Nesse documento, a ONU elenca o conjunto de 17 Objetivos de Desenvolvimento Sustentável (ODS)^(12)^. Entre eles, em face desta pesquisa, destacamos aquele que trata de garantir padrões de consumo e de produções sustentáveis (ODS 12), de modo a permitir ações que visam à redução significativa de geração de resíduos mediante prevenção, reciclagem e reuso. Nessa direção, outros ODS também, ainda que indiretamente, podem ser afetados pelo consumo e geração responsáveis de RSS, tais como saúde e bem-estar (ODS 3), água potável e saneamento (ODS 6) e ação contra a mudança global do clima (ODS 13).

Tratando-se de RSS, há também que se reconhecer a própria natureza complexa do sistema de saúde como estratégia fundamental para a geração e consumo responsáveis desses resíduos. Por sistema, entende-se a integração dinâmica entre as partes para o seu pleno desenvolvimento^(5)^. Nos sistemas de saúde de todo o planeta, tem-se que a enfermagem conforma a maioria dos recursos humanos, e seu destaque para o enfrentamento dessa realidade e de tantas outras não está restrito à expressão numérica desses profissionais, mas alcança significativo protagonismo em face dos múltiplos cuidados que realizam junto ao paciente, razão pela qual organizações globais reconhecem a profissão como estratégica para o alcance de parte dos ODS^(13-15)^.

A enfermagem é, portanto, profissão estratégica para alcançar práticas sustentáveis relacionadas ao consumo e geração responsáveis de RSS, sobretudo no ambiente hospitalar, tanto por ser a maior consumidora de recursos materiais para os cuidados em saúde quanto por ser a força motriz para planejar, implementar, supervisionar e avaliar ações resolutivas para o gerenciamento de RSS. Apesar disso, dados científicos sinalizam baixos níveis de conhecimento e *déficit* de práticas de preservação do meio ambiente e de seus recursos, bem como insuficientes práticas de gerenciamento de resíduos por parte dos profissionais de saúde^(16)^. Desse modo, a partir de uma perspectiva complexa, cabe questionar: como os profissionais de enfermagem percebem, em suas práticas profissionais no contexto hospitalar, ações relacionadas ao gerenciamento de RSS?

## OBJETIVOS

Compreender como o gerenciamento de RSS é desenvolvido por profissionais de enfermagem no ambiente hospitalar.

## MÉTODOS

### Aspectos éticos

Pesquisa foi aprovada em agosto de 2021 por Comitê de Ética e Pesquisa com Seres Humanos. Atendeu todas as exigências das Resoluções nº 466/12 e nº 580/18 do Conselho Nacional de Saúde. Para garantir o anonimato dos participandes do estudo, suas identidades foram preservadas a partir de indicações alfanuméricas. Desse modo, ao longo do artigo, foram designados como EN (enfermeiro) e TE (Técnicos de enfermagem), seguidos do número de suas respectivas entrevistas.

### Referencial teórico-metodológico

Para a interpretação dos dados e construção de conceitos, foi empregada a Teoria da Complexidade, na perspectiva de Edgar Morin. Para esta perspectiva paradigmática, valoriza-se a indissociabilidade das dimensões sociais, biológicas e econômicas dos fenômenos complexos. Nessa conjuntura, a sustentabilidade é concebida, em sua projeção sistêmica, mediante conexões que estabelece com a saúde, cultura, meio ambiente e sociedade, cujas interações entre cada parte afetam o ambiente circundante e também os próprios profissionais de saúde e pacientes^(5)^.

Para o processo analítico, utilizou-se a Teoria Fundamentada nos Dados (TFD), e foi adotada a vertente construtivista da escola “corbiniana” do método, que apresenta abordagem comparativa, perguntas constantes, amostragem teórica, elaboração e integração de conceitos, composta por três dimensões paradigmáticas, a saber: condições; ações-interações; e consequências^(17)^.

### Tipo de estudo

Pesquisa qualitativa, do tipo explicativa, por correlacionar subcategorias (princípios) em torno da categoria (conceito) para explicar a realidade investigada. Para a estrutura da pesquisa, foram empregadas as diretrizes da rede EQUATOR, notadamente o guia *COnsolidated criteria for REporting Qualitative research* (COREQ).

### Procedimentos metodológicos

Os participantes foram caracterizados a partir de formulário com dados sociodemográficos e profissionais. Para alcançar o objetivo da pesquisa, foram realizadas entrevistas semiestruturadas, cujas perguntas indutoras foram: o que significa, para você, gerenciamento de RSS? Como você percebe o consumo e a geração de RSS no hospital? Como você se percebe como profissional, integrante da equipe de enfermagem, em relação ao consumo e geração de RSS no hospital? A partir das respostas, perguntas circulares foram elaboradas, de modo a favorecer a apreensão consubstanciada dos dados. As entrevistas aconteceram em encontros individuais, em ambiente reservado, no próprio cenário de estudo, em horários previamente acordados e que não comprometessem as atividades laborais dos entrevistados.

### Cenário do estudo

Os dados foram coletados em um hospital universitário federal, na cidade do Rio de Janeiro, RJ, que apresenta diversas especialdiades clínicas, contendo cerca de 550 leitos. O estudo envolveu os setores de clínica médica e cirúrgica, por considerar que, nesses locais, independentemente das especialidades clínicas, há expressiva geração de RSS provenientes das assistências diretas e indiretas de enfermagem aos pacientes.

### Fonte de dados

Trata-se de amostragem por conveniência, em que os participantes foram convidados, presencialmente, no cenário do estudo. Não houve recusa de nenhum convidado ou desistência de participantes do estudo.

Participaram do estudo enfermeiros e técnicos de enfermagem, que compuseram, respectivamente, dois grupos amostrais. Foram incluídos profissionais com, no mínimo, um ano de experiência profissional na instituição, na assistência direta ao paciente como enfermeiro ou técnico de enfermagem no setor da clínica médica ou cirúrgica. Foram excluídos os profissionais do turno da noite, haja vista o entendimento de que a geração de RSS, no hospital, ser mais expressiva durante o dia, devido à rotina de cuidados/procedimentos de saúde.

A delimitação de dois grupos amostrais foi sustentada em hipótese de que, embora integrem uma só categoria profissional, enfermeiros e técnicos de enfermagem podem apresentar especificidades, tanto por aspectos deontológicos quanto por conhecimentos específicos.

### Coleta e organização dos dados

A coleta de dados ocorreu entre setembro e dezembro de 2021. As entrevistas foram gravadas em meio digital (áudio), e tiveram duração média de 30 minutos cada. Os pesquisadores realizaram teste piloto com três potenciais participantes, e não foi identificada a necessidade de ajuste do roteiro de entrevista. As entrevistas não foram repetidas com um mesmo participante. Foram empregados memorandos analíticos e reflexivos durante todo o percurso de coleta e análise, processo este que ocorre de forma simultânea na TFD. Esses memorandos permitem a formulação de hipóteses capazes de direcionar novas perguntas ou mesmo novos grupos amostrais, para que os investigadores compreendam onde o fenômeno está enraizado^(17)^. Ademais, os memorandos permitiram reflexões sobre o desenvolvimento dos conceitos, de modo que auxiliaram na compreensão sobre a saturação teórica dos dados, isto é, quando o conceito apresenta densidade teórica sustentada em suas respectivas subcategorias/princípios^(17)^. Cumpre destacar que o processo de saturação teórica fora discutido entre os pesquisadores antes da coleta de dados ser finalizada.

A coleta de dados ocorreu com dois pesquisadores enfermeiros, com *expertise* no método de coleta de dados e no referencial metodológico adotado, mediante competências desenvolvidas em outras pesquisas de morfologia metodológica semelhante, junto ao grupo de pesquisa ao qual estão vinculados. Não houve nenhum conflito de interesse relacionado aos profissionais ou ao cenário da pesquisa. Os pesquisadores não apresentam vínculo com o cenário da pesquisa.

Para facilitar a análise, organizar os dados, armazenar os arquivos, mapear os conceitos e elaborar relatórios de maneira rápida, simplificada e informatizada, os dados foram importados para o *software* NVIVO^®^ 12, após a transcrição no *Microsoft Office Word* ^®^ 2016. O NVIVO^®^ 12 é um *software* fundamental para os estudos de TFD, pois a expressiva quantidade de dados gerados no processo analítico é sistematicamente organizada com o *software* em questão^(18,19)^.

### Análise dos dados

Os dados foram submetidos à análise seguindo as etapas de codificação da TFD, a saber: aberta; axial; e integração seletiva. Na codificação aberta, os dados foram segmentados em partes distintas e rigorosamente examinadas, cuja comparação visou captar similaridades e diferenças entre os dados iniciais (códigos preliminares). Nesta etapa, os códigos são provisórios^(17)^.

A partir da análise comparativa dos dados, houve agrupamento destes por similaridades e diferenças. A partir desse processo, emergiram os códigos conceituais que são apresentados como uma representação abstrata de um fato, objeto ou ação que o pesquisador percebe como significativo. O agrupamento dos códigos conceituais por similaridades originou a categoria analítica^(17)^.

A codificação axial exige que o analista já tenha algumas subcategorias construídas, para relacioná-las à sua categoria, para, então, gerar explicações mais precisas e completas sobre o fenômeno investigado^(17)^. Na fase de integração, a categoria é refinada e desenvolvida a partir das abstrações teóricas de suas subcategorias.

Para este estudo, as subcategorias foram ordenadas a partir do modelo paradigmático da vertente da TFD construtivista da escola “corbiniana” do método^(16)^, que considera três componentes, a saber: condições, que trata dos fatores que influenciam o desenvolvimento do fenômeno; ações-interações, que tratam das estratégias de desenvolvimento e enfrentamento do problema; e consequências, que sinalizam as potenciais reações a partir das estratégias implementadas.

## RESULTADOS

Participaram da pesquisa dez enfermeiros e 22 técnicos de enfermagem, totalizando 32 participantes, dos quais 28 eram do sexo feminino e quatro eram do sexo masculino. A idade variou entre 27 e 58 anos, com uma média de 39 anos. O tempo de formação dos participantes apresentou média de 12 anos e nove meses, e média de tempo de trabalho no cenário da pesquisa de cinco anos e dez meses.

Sobre a formação dos participantes, dos 22 técnicos de enfermagem, dois relataram possuir ensino superior completo em enfermagem. No grupo dos enfermeiros, apenas um relatou não possuir pós-graduação *lato sensu.* Todavia, nenhum participante relatou ter realizado curso na área ou em aproximação com o gerenciamento de RSS.

Do processo analítico, emergiu a/o categoria/conceito “A multidimensionalidade do gerenciamento de resíduos de serviços de saúde na prática da enfermagem hospitalar”. Para tanto, a/o categoria/conceito foi estruturada/o a partir das/dos seguintes subcategorias/princípios, distribuídas/os no modelo paradigmático da seguinte maneira:

Condições: subcategoria 1: Ordens e desordens na geração e no descarte de resíduos de serviços de saúde no contexto hospitalar; subcategoria 2: Riscos e incertezas no descarte de resíduos de serviços de saúde.

Ações-interações: subcategoria 3: Gerenciamento do desperdício de materiais: a lógica não linear da geração e consumo de resíduos de serviços de saúde no contexto hospitalar; subcategoria 4: Do conhecimento à ação: a patologia do saber na formação dos profissionais de enfermagem e suas relações com o gerenciamento de resíduos de serviços de saúde.

Consequências: subcategoria 5: Produtos e produtores de si mesmos: o desafio do gerenciamento de resíduos de serviços de saúde durante a pandemia de COVID-19.

Cumpre destacar que os trechos de depoimentos dos participantes do estudo são, apenas, ilustrativos de cada subcategoria, haja vista que as mesmas foram desenvolvidas a partir de inúmeros códigos preliminares, resultantes de expressivo volume de dados oriundos das entrevistas transcritas.

### Subcategoria 1 - Ordens e desordens na geração e no descarte de resíduos de serviços de saúde no contexto hospitalar

Os resultados revelaram que a enfermagem se percebe produzindo expressiva quantidade de RSS, porém considera que é possível reduzir a geração desses resíduos através do gerenciamento racional dos recursos materiais sem afetar a qualidade da assistência.

*Mas a gente pode, sim, reduzir a quantidade do material utilizado, reduzir um pouco, vê o necessário, utilizar o necessário numa assistência.* (TE01)

Apesar de a geração de resíduos no contexto hospitalar ser uma preocupação dos profissionais de enfermagem, a redução da quantidade de resíduos gerados pode ser reconhecida como uma medida para a promoção da sustentabilidade percebida pelos próprios profissionais.

Os resultados demonstram também que essa alta produtividade de RSS, no ambiente hospitalar, pode ser influenciada pela escassez de recursos materiais de qualidade para a assistência à saúde. Nessa conjuntura, os profissionais precisam encontrar diferentes maneiras de lidar com a falta de equipamentos e suprimentos adequados para fornecer assistência de qualidade aos pacientes. Adaptar-se às circunstâncias e encontrar soluções criativas para superar a falta de recursos, diante das limitações enfrentadas no serviço, foram desafios sinalizados pelos profissionais de enfermagem nessas situações. Dessa realidade, há o aumento da geração de RSS.

*Então, na nossa realidade, a gente faria, por exemplo, o curativo, a gente usaria a cuba para poder jogar um soro, aí fazer o curativo depois* [...] *aqui a gente está até sem a autoclave, então não tem como ficar utilizando uma cuba, uma bacia, aí é o que? É* [...] *fralda ou então, compressa, então a produção de lixo, um lixo que não produziria dessa forma, se tivesse a cuba, a bacia, a gente acaba produzindo, porque não tem como*. (TE08)

Os resultados destacaram, ainda, a necessidade da relação precisa de todos os materiais utilizados na prestação de assistência como forma de controlar os insumos. Nessas circunstâncias, processos de trabalho simples, como especificar o número de pacotes de gaze, luvas estéreis e outros materiais a serem utilizados nos diversos procedimentos executados pela enfermagem, são apontados como relevantes.

[...] *quando você vai relatar, por exemplo, curativo que você realizou, você tem que anotar quantos pacotes de gaze você utilizou, quantas luvas estéreis, se foi soro, se foi clorexidina alcoólica; tudo isso tem que ser relatado.* (EN05)

Destarte, os profissionais de enfermagem reconhecem o gerenciamento de RSS como demanda do processo de trabalho que realizam e que devem primar pela redução da produção desses materiais. Nesse sentido, em seus significados, pontuam o planejamento do material para a assistência de enfermagem como ponto inicial para o cuidado sustentável.

Os dados revelaram, também, as condições estruturais e os recursos oferecidos pelo contexto de trabalho para o adequado gerenciamento dos resíduos e as barreiras enfrentadas pelos profissionais no cotidiano hospitalar.

A rotina e as demandas excessivas de cuidados impulsionam acelerado ritmo de trabalho e escassez de tempo, que afetam os processos de tomada de decisão, sobretudo aquelas que solicitam agilidade do profissional, como separar material em quantidade maior que o necessário para a realização de determinado cuidado, gerando desperdício de materiais.

*Eu acho que a correria do dia a dia, a falta de tempo* [...] *você sai, vou abrir, porque senão vou perder tempo se eu deixar para solicitar para alguém caso eu precise de mais*. (EN07)

Os dados revelam que a celeridade do trabalho e a consequente escassez de tempo favorecem a segregação inadequada dos RSS. Ademais, os profissionais sinalizaram saber que a pressa e a falta de atenção no momento de descartar os resíduos podem levar a equívocos, resultando em uma disposição inadequada e potencialmente perigosa dos materiais utilizados em procedimentos de saúde. Essa segregação equivocada compromete a eficácia dos processos de coleta, transporte e tratamento, aumentando os riscos de contaminação ambiental e colocando em perigo a saúde dos profissionais envolvidos, bem como a comunidade no entorno.

*Não dá nem tempo nem de querer separar. É muito difícil, tem dias que a gente fica parecendo um robô às vezes*. (TE21)

*Acaba fazendo errado, por fazer tudo correndo e ganhar tempo. Eu acho que sempre é questão do tempo, parar para fazer as coisas no tempo certo, eu acho que reduzem bastante os ricos de fazer o descarte incorreto*. (TE12)

Cumpre destacar que a hipótese que direcionou a composição de dois grupos amostrais desta pesquisa, como enfermeiros e técnicos de enfermagem, pode apresentar especificidades, tanto por aspectos deontológicos quanto por conhecimentos específicos, e não foi corroborada pelos dados, visto que não houve especificidades nos resultados em relação aos grupos supracitados.

### Subcategoria 2 - Riscos e incertezas no descarte de resíduos de serviços de saúde

Essa subcategoria revela, a partir da percepção dos profissionais de enfermagem, como, em suas próprias práticas, são realizados o descarte e a segregação dos RSS. Desse modo, revelaram incoerências desses processos quando ocorre de acordo com os participantes. Essa realidade é complexa por ser influenciada por diversos fatores, conforme exemplificado nos trechos seguintes:

*A bolsa de sangue, né?! Após a transfusão, hoje vai no lixo comum. Pelo que a gente sabe, assim, pode ser que esteja errado, primeiro você filtra, mas, às vezes, a gente tem dúvida.* (TE18)

*Às vezes, jogam sem querer, porque passa desapercebido, por mais que seja uma coisa importante* [...] *todo mundo bate na mesma tecla: “descartar agulha é no descarpack”, mas, de vez em quando, a gente pega uma agulha na lixeira.* (TE12)

[...] *eu já vi descarte de material perfurocortantes sendo jogadas no lixo comum, no infectante, coisas assim que são grosseiras* [...] *descarte, por exemplo, de material, de fluído de material contaminado em lixo comum*. (EN03)

Apesar de os resíduos comuns representarem, no setor de saúde, a maior proporção em relação aos resíduos perigosos, como os perfurocortantes, estes últimos são os de maior preocupação manifestada pelos profissionais de enfermagem. Em meio aos riscos, incertezas e ilusões da complexidade imbuída nessa conjuntura, os profissionais pesquisados demonstraram compreender os riscos próximos de si quando significam a importância do descarte de RSS de maior periculosidade em seus contextos laborais.

*Esse cuidado é todo pela gente, para que a gente não se fure, para que não tenhamos um acidente e também a separação desse lixo, da contaminação, do que é vidro, ampola, vidro mesmo de medicamento, antibióticos. Tem que ter uma separação desse lixo.* (TE18)

Ademais, entre os fatores multidimensionais relacionados ao gerenciamento de RSS, a disposição de recursos materiais, infraestrutura e fluxos organizacionais foi pontuada pela equipe de enfermagem. Nessa realidade, dispositivos e medidas supostamente simples, como a disponibilidade de lixeiras para a segregação de resíduos infectantes e estratégias didáticas pautadas em informações eficientes para os pacientes e seus acompanhantes, foram sinalizadas como capazes de reduzir chances de haver mistura de resíduos infectantes e comuns, incluindo restos de comida e equipamentos.

*Não sei te dizer, não sei se falta lixeira, que antes todas as enfermarias tinham duas lixeiras, a do lixo comum, do lixo hospitalar e dos perfurocortantes, mas o lixo comum geralmente não tem. A comida, a gente vai jogar junto com os equipos* [...] *que é onde a gente descarta o soro, os equipos, fralda, etc. Então, meio que fica tudo junto, mas, assim, é em algumas enfermarias, outras têm, não sei porque, não sei o que houve.* (TE09)

*Às vezes, nem só do técnico, nem só do profissional, mas também existem outras pessoas que ficam na enfermaria, que não têm esses conhecimentos. Os acompanhantes, por exemplo, eles não têm esses conhecimentos* [...] *se ele tem que jogar em qualquer uma das duas, para eles, aquilo dali é lixo, é a lixeira, eles não irão saber*. (TE17)

A realidade supracitada é corroborada não apenas pelos técnicos de enfermagem, mas também pelos enfermeiros.

*O que a gente observa é que todo mundo usa as duas para tudo. Você abre o lixo de infectante, tem comida dentro, entendeu? Não tem só coisa que devia estar ali dentro, então a gente vê que não funciona na enfermaria*. (EN05)

*Olha, aqui no hospital, apesar de haver lixeiras brancas, com separação de resíduos infectantes e não infectantes, eu acho que não são muito bem* [...] *a separação não é legal, porque você abre a lixeira ali do infectante, está tudo* [...] *está comida, fralda, está garrafa de água*. (EN08)

Outro fator relacionado à multidimensionalidade do gerenciamento de RSS está no conhecimento dos profissionais de enfermagem em relação ao Plano de Gerenciamento de Resíduos de Serviços de Saúde.

*O Plano* [Plano de Gerenciamento de Resíduos de Serviços de Saúde] *não conheço, não. Aqui, o que a gente aprendeu foi na prática*. (TE14)

Além das questões relacionadas ao conhecimento, as atitudes dos profissionais também são por eles reconhecidas como condição interveniente para os desafios da enfermagem em direção ao gerenciamento de RSS. Nesse ínterim, a falta de estímulo à separação de materiais recicláveis influencia atitudes em prol das práticas sustentáveis.

[...] *não recebi nenhuma orientação de que haja lugar para ter descarte de lixos descartáveis, não! Por exemplo, as garrafas de água que a gente usa* [...] *podem ser recicladas e mesmo assim vão para o lixo normal, e isso desanima.* (EN08)

### Subcategoria 3 - Gerenciamento do desperdício de materiais: a lógica não linear da geração e consumo de resíduos de serviços de saúde no contexto hospitalar

Essa subcategoria demonstra como a geração dos resíduos é influenciada pelo gerenciamento dos desperdícios dos materiais hospitalares. Ademais, os participantes consideraram que uma assistência de enfermagem sem planejamento de recursos pode desencadear o uso inapropriado dos insumos e, desse modo, impulsionar a geração de desperdícios. Para os enfermeiros, a geração de RSS é inevitável, em contrapartida, o desperdício é gerenciável.

*Eu observei hoje, né?! Gaze, por exemplo, pacote de gaze é aberto, e depois pegaram tudo e jogaram fora, porque já estava aberto*. (EN05)

*No outro dia, em uma enfermaria que fechou por algum problema técnico mesmo, os pacientes foram para outros andares, mas estava com tanta coisa na enfermaria, umas sete almotolias, tudo ia para o lixo, o que eu ia fazer? Não posso botar em outro paciente. Então acaba, assim, com esse desperdício*. (EN01)

Para os profissionais de enfermagem, à medida que precisam improvisar em decorrência da escassez de recursos materiais, as medidas adotadas geram mais resíduos, haja vista a necessidade de utilização de maior quantidade de insumos que passam a ser adaptados para que desenvolvam os cuidados ao paciente da melhor maneira possível. Desse modo, os dados sinalizaram que a suposta economia de recursos materiais manifestada no *déficit* destes, apresenta, na verdade, agravantes econômicos em decorrência da improvisação necessária às práticas de cuidados. Agravam-se, devido a essa realidade, os impactos no meio ambiente e na saúde dos profissionais de saúde e dos pacientes.

*Então, quando tem o material adequado, não, mas quando o hospital está em falta, a gente tem que improvisar com outras coisas, aí que leva o desperdício. Se tiver o material certinho, a gente não desperdiça.* (TE10)

Nem sempre os RSS apresentam origem a partir de cuidados diretos ao paciente. Um dado recorrente da pesquisa sinalizou que os profissionais de enfermagem apontam o excessivo desperdício de papel A4, por exemplo.

*Cada vez há mais impressos, o que não tinha aqui e que já deveria ter diminuído com o avanço da tecnologia. Às vezes, surge um papel, por exemplo, todo paciente que vai ser encaminhado para algum tipo de exame. Antes, a gente fazia o encaminhamento na evolução corrida, agora eles inventaram uma folha de transferência, então é mais um papel para todo paciente que vai para qualquer lugar. Então, se a gente tem 27 pacientes, a gente fez umas dez transferências. São dez papéis, entendeu? Eu acho um absurdo, tem que pensar não só no custeio, mas na produção de lixo.* (EN02)

Portanto, o gerenciamento do desperdício de materiais foi considerado prática importante que deve ser apreendida no cotidiano hospitalar dos profissionais de enfermagem, de modo a possibilitar medidas positivas na economia da saúde em sintonia com as práticas sustentáveis.

### Subcategoria 4 - Do conhecimento à ação: a patologia do saber na formação dos profissionais de enfermagem e suas relações com o gerenciamento de resíduos de serviços de saúde

Essa subcategoria revela que o conhecimento técnico especializado e habilidades práticas são condições para o gerenciamento de RSS. Para tanto, os profissionais de enfermagem consideraram que a sua equipe deve estar preparada para lidar com resíduos diversos, como materiais perfurocortantes, infectantes, produtos químicos, medicamentos, entre outros que possam apresentar risco à saúde deles próprios, dos pacientes e do meio ambiente.

Ainda que as normas e legislações exijam que os profissionais de enfermagem devam estar aptos às adequadas práticas no manejo dos RSS, promovendo a segurança e a eficácia no seu gerenciamento, os resultados revelaram insuficiência de conhecimentos, cuja realidade é atribuída pela escassez de capacitação oferecida sobre a temática. Para os participantes, essas medidas podem influenciá-los na identificação e classificação correta dos resíduos gerados pelos serviços de saúde, seguindo as normas e regulamentações nacionais e internacionais.

*Falaram sobre gerenciamento de resíduo, mas eu acho que, no treinamento de uma empresa de limpeza, eles falaram sobre isso, mas bem pouca coisa também. Até os cursos não ensinam. Eu venho de uma época bem antiga.* (TE02)

A natureza complexa dos resultados direcionou o entendimento de que o saber-fazer da enfermagem no gerenciamento de RSS também é influenciado pela inserção ou ausência da temática ambiental nas grades curriculares, tanto na formação de nível médio quanto na de ensino superior. Para os participantes do estudo, pouco ou nenhum tipo de abordagem sobre o assunto fez parte da formação profissional. Apesar do exposto, a práxis da enfermagem direcionada às práticas sustentáveis relacionadas ao processo de geração e consumo de RSS deve ser fundamentada em conhecimentos técnicos sobre os tipos de resíduos, suas características e classificação, bem como por habilidades que permitam manusear esses materiais com segurança e prevenir riscos à saúde e ao meio ambiente.

### Subcategoria 5 - Produtos e produtores de si mesmos: o desafio do gerenciamento de resíduos de serviços de saúde durante a pandemia de COVID-19

Os resultados da pesquisa apresentaram pertinência contextual ao momento histórico de enfrentamento da pandemia de COVID-19, realidade esta que influenciou, também, de acordo com os participantes do estudo, a geração de RSS. Desse modo, com o aumento da demanda por serviços de saúde, houve também um aumento significativo na geração de resíduos, o que tornou o gerenciamento desses materiais ainda mais desafiador.

*A preocupação com os EPIs aumentou, mas aumentou o uso de material, aumentou muito capote, luva, máscara* [...] *materiais que eram mais importantes na COVID-19 tiveram aumentou significativo, por uma questão de demanda mesmo.* (TE06)

Os profissionais de enfermagem destacaram que o período pandêmico potencializou uma prática que já ocorre no cotidiano do trabalho na área da saúde. Naquela conjuntura, porém, o desperdício e descartes inadequados de equipamentos de proteção individual, suprimentos e outros recursos foram ainda mais expressivos, de acordo com os membros da equipe de enfermagem.

*Eu acho que teve muito desperdício* [...] *falta de conhecimento da doença, uma coisa nova ninguém conhece, não conhece até hoje, né?! Pegou todo mundo despreparado, aí teve muito gasto de material, a gente vê muita coisa*. (TE04)

*O consumo de EPI foi intensificado e também se viu muito descarte impróprio, não apenas nos hospitais, mas em todo lugar era possível observar máscaras jogadas em lugares impróprios, por exemplo.* (EN09)

Em conformidade com os dados apresentados pelos participantes e sob o enfoque da complexidade que conduziu este estudo, a pandemia recente apenas revelou a importância de uma consciência sistêmica que foge do contexto dos profissionais de saúde e afeta toda a sociedade. Desse modo, a consequência prospectada, a partir do que sinalizaram os dados nas subcategorias anteriores, parece orbitar na importância de uma formação cidadã e contextualizada capaz de reconhecer e valorizar a necessidade de práticas responsáveis no consumo, geração e descarte de resíduos.

## DISCUSSÃO

A perspectiva dos profissionais de enfermagem sobre a forma como esses estão implicados no gerenciamento de RSS corrobora o entendimento de que o contexto hospitalar é foco de atenção para a problemática que envolve a geração de RSS, e essa realidade pode apresentar especificidades em relação às políticas/culturas institucionais de gestão dos hospitais, conforme indica o estudo realizado em hospitais privados, cujos dados indicaram geração de 8,22 (6,39-10,02) kg/leito/dia, demonstrando que tendem a ser maiores geradores do que os hospitais públicos^(20)^.

Em conjuntura sociopolítica e econômica, sabe-se que o Brasil é um país continental, em desenvolvimento e industrializado, e esta configuração também deve ser contemplada quando se trata da geração de RSS, sobretudo ao se considerar o objeto deste estudo, com a enfermagem sendo percebida como importante fator interveniente para o gerenciamento de RSS, posto se tratar, em conformidade com outras realidades globais, do principal contingente de recursos humanos do setor de saúde. Nesse sentido, tem-se que a média de RSS gerada em países de alta renda geralmente varia entre 2 e 4 kg/leito/dia, o que é menor em comparação a países de renda média-alta e baixa, variando entre 4 e 6 kg/leito/dia, o que pode ser devido ao fato de países de alta renda apresentarem melhores políticas de gestão, tecnologias de descarte mais avançadas, bem como autoridade reguladora competente e trabalhadores treinados, em comparação com países de renda média-baixa^(21)^.

Diante do exposto, tal qual sinaliza o princípio circuito-recursivo da complexidade, cujo processo orienta que os produtos e efeitos são, paralelamente, causas e produtores daquilo que os produziu^(5)^, tem-se que a relevância da enfermagem nos sistemas de saúde pode conformar força motriz valorosa em direção às adequadas condutas para o gerenciamento de RSS, sobretudo a partir de medidas apropriadas para reduzir a geração de RSS no contexto hospitalar em suas operações diárias, como o controle dos insumos de forma sistematizada, com informações sobre o volume de RSS gerados pelos profissionais de saúde^(22)^. Todavia, em conformidade com o princípio circuito-recursivo^(5)^, o gerenciamento dos RSS pode ser comprometido quando a enfermagem não assume o seu protagonismo nesse processo, porém, de acordo com os dados da nossa pesquisa, essa realidade não necessariamente depende apenas da enfermagem, mas também das condições de trabalho, que são afetadas pela sobrecarga dos profissionais e pelo *déficit* de recursos materiais.

A infraestrutura foi sinalizada como fator que influencia o gerenciamento de RSS. Nesse sentido, a respeito das condições encontradas para o descarte de RSS, foi revelado, em pesquisa, que a maioria dos inquiridos, 358 (90,86%) dos centros de saúde e 133 (96,38%) dos hospitais pesquisados, indicou que as suas instalações tinham contentores separados para resíduos perigosos e não perigosos; e 61 (15,48%) dos inquiridos dos centros de saúde e 29 (21,01%) dos hospitais indicaram que os recipientes de RSS não estavam claramente marcados ou rotulados. No mesmo estudo, 241 (67,3%) usaram as lixeiras existentes para colocar resíduos nas instalações de saúde pública, e 32 (23,19%) dos hospitais indicaram que os recipientes de RSS não estavam localizados em locais apropriados onde poderiam ser necessários^(23)^.

Com relação à segregação de resíduos recicláveis em hospitais, a literatura sinaliza a necessidade de se implementar políticas de gestão de resíduos sólidos que estabeleçam as diretrizes e os procedimentos adequados para a segregação, coleta, transporte, armazenamento, tratamento e disposição final dos resíduos gerados^(24,25)^. A geração de resíduos destinados à reciclagem, de acordo com dados de pesquisa, foi de 0,72 (0,45- 1,02) kg/leito/dia, o que representa apenas 12% do total de resíduos gerais e demonstraram como um bom gerenciamento de resíduos permite a implantação da coleta seletiva em ambientes hospitalares^(22)^.

A avaliação constante da quantidade de materiais utilizados nos procedimentos, considerando a possível redução sem afetar a qualidade da assistência, bem como o uso de materiais reutilizáveis, com a substituição de materiais descartáveis por reutilizáveis, pode favorecer a economia e a redução da quantidade de resíduos gerados. Depreende-se, dessa realidade, por exemplo, o uso de pratos, copos e talheres reutilizáveis em vez de descartáveis, em conjunto com o incentivo da implementação de um programa de reciclagem que viabilize a separação dos recicláveis, fortaleça a educação permanente em saúde e que aborde a temática sob uma lógica sistêmica. Além disso, faz-se imprescindível o controle de estoque de material que favoreça a diminuição de compras excessivas ou insuficientes para os serviços, assim como a redução do descarte de medicamentos vencidos^(22)^.

Outro aspecto apontado no estudo e que influencia o gerenciamento de RSS está no conhecimento sobre essa temática. Desse modo, em relação ao conhecimento da existência do Plano de Gerenciamento dos Resíduos de Serviços de Saúde, pesquisa cita que 71,4% dos enfermeiros e 40% dos técnicos de enfermagem afirmam não saber se a instituição conta com o referido Plano. Essa taxa é ainda menor quando comparada ao conhecimento dos enfermeiros sobre a existência de uma Comissão de Controle de Infecção Hospitalar (CCIH), bem como de Comissão Interna de Prevenção de Acidentes (CIPA): 86% dos técnicos e 85% dos enfermeiros^(24)^.

A fragilidade da formação de enfermeiros e técnicos de enfermagem, apontada pelos participantes do estudo, em relação aos conhecimentos sobre gerenciamento de RSS, sugere relação direta com a necessidade de se pensar uma educação que valorize a complexidade no decurso do processo formativo do enfermeiro, bem como nas práticas relacionadas à educação permanente em saúde. Para tanto, os dados reforçaram a necessidade de que as instituições de ensino e de saúde oportunizem estratégias que contextualizem a sustentabilidade ambiental em perspectivas aproximadas da prática profissional da equipe de enfermagem.

Embora a humanidade tenha atravessado a principal crise sanitária global dos últimos anos, com a pandemia de COVID-19, essa realidade recente demonstrou, em larga escala, a necessidade de haver melhores investimentos na formação dos profissionais e a devida atenção dos governos e instituições de ensino e de saúde para as questões ambientais, notadamente àquelas relacionadas ao consumo e descarte responsáveis de resíduos.

Ainda se tratando do período pandêmico recente, tem-se que tal contexto potencializou práticas inadequadas para o consumo, geração e descarte de RSS, bem como *déficits* de recursos materiais e de infraestrutura relacionados a essa problemática, em paralelo à expansão de fluxos de hospitalização de pacientes. Nessa conjuntura, equipamentos de proteção individual, utilizados pelos profissionais de saúde, para o tratamento de pacientes doentes e as medidas de segurança preventivas obrigatórias, como máscaras faciais e luvas, levaram a um aumento substancial no acúmulo de resíduos em todo o mundo. Na China, por exemplo, cerca de 240 toneladas de RSS foram descartadas na pandemia diariamente; antes essa quantidade correspondia a 40 toneladas em dias normais^(26)^. Todavia, a problemática relacionada à geração excessiva de resíduos não decorre apenas de crises sanitárias globais, mas também do processo natural de crescimento populacional e consequente demanda por recursos materiais, consumo, geração e descarte de resíduos. Nessa conjuntura, como exemplo, tem-se que, mesmo antes da pandemia de COVID-19, a geração de RSS na Índia havia aumentado de 559 para 613 toneladas/dia entre 2017 e 2019, e, no decorrer da luta contra a pandemia, o país gerou cerca de 850 toneladas de RSS por dia^(27)^.

### Limitações do estudo

A realidade investigada pode ter influenciado os sistemas de significados dos participantes em relação ao gerenciamento de RSS, especialmente aqueles relacionados às condições de recursos materiais, infraestrutura e cultura organizacional. Isto porque hospitais universitários podem apresentar especificidades não observadas em hospitais privados, por exemplo. Em perspectiva epistemológica, declara-se como limitação do estudo a importância de validação dos resultados junto a *experts*, como ocorre em parte dos estudos com TFD.

### Contribuições para as áreas da enfermagem, saúde ou políticas públicas

Embora os profissionais de enfermagem se reconheçam como capazes de influenciar a redução de RSS sem, contudo, afetar a qualidade da assistência, sinalizaram a importância de processos de trabalho que permitam reconhecer a multidimensionalidade envolvida no gerenciamento dos RSS. Ademais, sob a perspectiva da enfermagem, o estudo sinaliza que o *déficit* de recursos materiais impulsiona processos laborais pautados em improvisações criativas para o suprimento desses recursos. Essa realidade, acrescida da sobrecarga dos trabalhadores investigados, impulsiona a geração de RSS.

O estudo sinaliza a fragmentação dos conhecimentos da enfermagem, sob perspectiva dos profissionais da área, em relação aos conhecimentos sobre gerenciamento de RSS. Todavia, os participantes destacam que o conhecimento técnico-especializado e habilidades práticas são condições para o gerenciamento de RSS. Nessa conjuntura, os profissionais de enfermagem identificaram que a fragilidade de conhecimentos sobre a temática pode decorrer do *déficit* de abordagens na formação regular (graduação), bem como no serviço de saúde (capacitações). Depreende-se dessa realidade a importância de valorização da temática relacionada ao gerenciamento de RSS na formação dos profissionais de enfermagem.

## CONSIDERAÇÕES FINAIS

O gerenciamento de RSS, no ambiente hospitalar, é realizado pelos profissionais de enfermagem em uma perspectiva complexa, cujas dimensões envolvem fatores intervenientes para o seu desenvolvimento, como estratégias e consequências que vão desde a segurança do profissional e do paciente até a saúde da população e preservação do meio ambiente.

A escassez de recursos materiais, associada à necessidade de improvisação com os insumos disponíveis, foi apontada como realidade que influencia a geração excessiva de RSS. Ademais, as demandas excessivas de cuidados, associadas ao ritmo acelerado de trabalho, afetam os processos de tomada de decisão e, com isso, potencializam chances de erro dos profissionais para o desperdício de materiais e descarte inadequado dos RSS. Apesar disso, os profissionais de enfermagem demonstraram compreender os riscos próximos de si quando significam a importância do descarte de RSS de maior periculosidade em seus contextos de trabalho.

Para a equipe de enfermagem, sua práxis relacionada ao gerenciamento de RSS é influenciada pela inserção ou ausência da temática ambiental em seus processos formativos, bem como pela insuficiência de oportunidades que discorram sobre RSS em ações relacionadas à educação permanente em saúde.

Os enfermeiros e técnicos de enfermagem reconhecem que a pandemia de COVID-19 apenas intensificou um problema crônico relacionado ao consumo, geração e descarte de RSS, de modo que reconhecem a importância de uma consciência sistêmica capaz de valorizar a importância de práticas responsáveis e sustentáveis em relação aos RSS.
